# Improving diagnostic stewardship of *Streptococcus pneumoniae* urine antigen testing among cancer patients

**DOI:** 10.1017/ash.2026.10885

**Published:** 2026-09-21

**Authors:** Justin Laracy, Athanasios Gatsis, Judy Yan, Rich Kodama, Mini Kamboj

**Affiliations:** 1 Memorial Sloan Kettering Cancer Center, USA; 2 Aristotle University of Thessaloniki School of Medicine: Aristoteleio Panepistem, Greece

## Abstract

We evaluated the utility of nearly twelve thousand *Streptococcus pneumoniae* urine antigen tests at a tertiary cancer center. Overall, test positivity rate was low. Testing during hospital days 0–2 yielded a greater positivity rate than testing at hospital 3 or beyond (1.2% vs 0.89%, *P* = .15). This trend was preserved among patients with neutropenia (1.2% vs 0.68%; *P* = .30) and those who had received cellular therapy within the preceding year (1.3% vs 0.34%; *P* = .11).

## Introduction

Hospital-acquired pneumonia (HAP) is defined as pneumonia that was not incubating at the time of admission and develops ≥48 hours after admission. HAPs are among the most common type of healthcare-associated infection and carry a high degree of associated morbidity and healthcare cost.^1^ As a result, considerable diagnostic and therapeutic attention is directed toward identifying causative pathogens in hospitalized patients with suspected pneumonia. While a broad range of organisms can cause pneumonia in the inpatient setting, the epidemiology of HAP differs substantially from that of community-acquired pneumonia (CAP) wherein *Streptococcus pneumoniae* remains a leading causative agent.^2^ By contrast, *S. pneumoniae* is an uncommon cause of HAP, accounting for approximately 5% of microbiologically defined HAP episodes in a systematic review of the available literature.^3^

The Binax NOW® is an immunochromatographic membrane assay for the detection of pneumococcal antigen in the urine with a well-established utility in the diagnostic evaluation of CAP.^4^ However, its diagnostic value in patients with HAP remains uncertain. Guidelines from the Infectious Diseases Society of America and American Society of Microbiology recommend the *S. pneumoniae* urine antigen test as an adjunctive diagnostic tool for the evaluation of HAP in immunocompetent hosts but make no specific comment on its use in immunocompromised patients.^5^ Additionally, recent contributions to the critical care literature also support use of the *S. pneumoniae* urine antigen test for the evaluation of HAP.^6,7^

In the present study, we retrospectively evaluate the yield of *S. pneumoniae* urine antigen testing among all patients at a tertiary care cancer center to 1. describe the *S. pneumoniae* urine antigen test yield in the inpatient and ambulatory settings and 2. propose a *S. pneumoniae* urine antigen test diagnostic stewardship framework among hospitalized oncology patients.

## Methods

### Study population

Memorial Sloan Kettering Cancer Center (MSKCC) is a 514-bed tertiary cancer center in New York City with approximately 25,000 admissions and 173,000 annual patient days. From January 1, 2020, to December 31, 2024, all *S. pneumoniae* urine antigen tests (UATs) from all adult and pediatric patients were included in the study. During the study period there were no institutional restrictions or clinical decision support tools in place to influence ordering of the test. Although the limited role of *S. pneumoniae* in HAP provides the rationale for examining late-hospitalization testing, the present study evaluates the yield of *S. pneumoniae* UAT among all patients undergoing testing rather than restricting the cohort to patients meeting clinical criteria for pneumonia. This study was conducted under an IRB-approved protocol that included waivers of informed consent and HIPAA authorization.

### Study method

The primary outcome analyzed was positivity rate by certain cancer-specific host factors (absolute neutrophil count (ANC) and prior cellular therapy) and hospital admission status at time of sample collection (outpatient, hospital days 0–2, and hospital days ≥3). A secondary outcome of clinically significant infection for positive hospital-acquired samples was ascertained by blinded independent review of the electronic medical record by two infectious diseases physicians, with a third physician adjudicating cases of disagreement.

At the study institution, urine samples were collected according to the manufacturer’s instructions for use.^4^ Any patient with an ANC ≤ 500 k/µL on or within 3 days of the urine sample collection date was categorized as neutropenic.

### Statistical analysis

Baseline demographic and clinical characteristics for all study patients were reported as absolute frequency and percentage. Positivity rates between hospital days 0–2 and hospital days ≥3 were compared using two-sided Fisher’s exact tests.

## Results

### Baseline characteristics of study cohort

During the study period, there were a total of 11,594 *S. pneumoniae* UATs collected from 8,172 unique patients. Slightly more than half of samples were collected from patients who were ≥65 years of age and from males. Most samples were collected in the inpatient setting. The underlying cancers were 2,565 (31.4%) patients with hematologic malignancy, and 5,566 (68.1%) with solid tumors. A total of 1,319 (11.4%) samples were collected from patients who had received hematopoietic stem cell transplant or chimeric antigen receptor T-cell therapy within the prior year, and 1,491 (12.9%) were collected from patients with neutropenia (Table 1). 522 samples (4.5%) were collected from patients who were requiring mechanical ventilation at the time of sample collection.

**Table 1.** Demographics and baseline characteristics of study patients Table 1 long description.

Characteristic	n (%)
Age ≥ 65 y	6,490 (56.0%)
Age < 65 y	5,104 (44.0%)
Age 0–18 y	63 (0.5%)
Male sex	6,173 (53.2%)
Female sex	5,421 (46.8%)
Inpatient^a^	10,511 (90.7%)
Outpatient^a^	1,083 (9.3%)
Cancer type	
Hematologic malignancy	2,565 (31.4%)
Solid tumor	5,566 (68.1%)
Other	41 (0.5%)
Neutropenic (yes)^b^	1,491 (12.9%)
Neutropenic (no)^b^	10,103 (87.1%)
BMT or CAR-T (yes)^c^	1,319 (11.4%)
BMT or CAR-T (no)^c^	10,275 (88.6%)

BMT, bone marrow transplant; CAR-T, chimeric antigen receptor T-cell.

a Patient location at time of urine sample collection.

b Neutropenic if absolute neutrophil count was ≤500 k/µL on or within 3 days of urine collection date.

c BMT and CAR-T therapy combined. Service dates are prior to urine sample collection and are within 1 year of sample date.

### Binax NOW positivity rate by location and population

The Binax NOW positivity rate was greatest during hospitals days 0–2 at 1.2% whereas the positivity rates in the outpatient setting and during hospital days ≥3 were 0.92% and 0.89%, respectively. This pattern was maintained among the transplant and neutropenic patient subgroups. Specifically, the positivity rates among patients with neutropenia and after cellular therapy were greatest in the earlier stage of admission (Table 2). Fisher’s exact test comparing hospital days 0–2 and hospital days ≥3 did not find significant differences in UAT positivity rate. Positivity rate among mechanically ventilated patients was 1.72%

**Table 2.** Positivity rate of *Streptococcus pneumoniae* urinary antigen test via Binax NOW by patient location and host immune status Table 2 long description.

	Outpatient	Hospital days 0–2	Hospital days ≥3	*P* value^a^
Positivity rate overall	10/1083 (0.92%)	96/7695 (1.2%)	25/2816 (0.89%)	.15
Positivity rate during the case of neutropenia	0/22 (0%)	9/731 (1.2%)	5/738 (0.68%)	.30
Positivity rate within 1 year of cellular therapy	1/115 (0.87%)	8/618 (1.3%)	2/586 (0.34%)	.11

a *P* values represent Fisher’s exact tests comparing hospital days 0–2 with hospital days ≥3.

### Clinical review of positive Binax NOW tests detected during hospital days ≥3

A total of 25 unique patients had a first-time positive *S. pneumoniae* UAT after hospital day 3. The number of days from admission to test positivity ranged between 3–79 (median 8 d). Three patients had *S. pneumoniae* isolated in culture (all 3 in blood) either upon admission or preceding admission, and five additional patients had clinical features of *S. pneumoniae* infection at the time of admission, with delayed UAT testing after hospital day 3. Of the remaining 17 UATs representing patients without culture-confirmed *S. pneumoniae* infection or signs/symptoms of clinic infection upon admission, blinded review of the electronic medical record by three infectious disease physicians found that only one case represented true hospital-acquired infection. The positive UAT did not change clinical management in any of these patients except for one patient where the positive test result led to antibiotic de-escalation from piperacillin-tazobactam to ceftriaxone after the UAT returned positive.

## Discussion

In this retrospective study assessing the utility of the *S. pneumoniae* UAT among patients at a tertiary cancer center, we found that the overall test yield was low across all settings. The positivity rate was highest in the early stage of admission and then declined among samples collected beyond day 3 and remained low across the various high-risk immunocompromised subgroups. While these findings are consistent with the established epidemiology of pneumonia in hospitalized patients, they support limiting use of the *S. pneumoniae* UAT in immunocompromised patients past hospital day 3.

While guidelines from the IDSA mention the use of *S. pneumoniae* UAT in the work-up of hospital-acquired or ventilator-associated pneumonia (HAP-VAP), the 2016 guidelines for the management of HAP-VAP make no comment on its use.^8^ Evidence to support pneumococcal UAT for HAP diagnosis is limited, and the recommendation remains tentative.^5^ It is important to note that the pathogenesis of pneumococcal pneumonia is typically community-acquired with progression of infection prior to hospitalization, especially due to drug-resistant strains, as the probable explanation of late-onset HAP that is attributed to this pathogen.^9,10^ Together, these epidemiological and biological considerations suggest that diagnostic testing targeting pneumococcal infection is likely to have the greatest diagnostic benefit early in the course of hospitalization.

Furthermore, the clinical utility of pneumococcal UAT in this setting is further limited by the treatment implications of the test results. Not uncommonly in clinical practice when there is substantial diagnostic uncertainty, empiric antibiotic regimens commonly used for HAP-VAP typically include broad-spectrum β-lactam agents to target *Staphylococcus aureus* and gram-negative pathogens, which universally provide reliable activity against *S. pneumoniae*, and a negative UAT may not be consequential for antibiotic de-escalation. Collectively, these findings suggest that continued use of pneumococcal UAT for HAP cases may contribute to diagnostic overuse without providing meaningful clinical benefit.

The overall UAT positivity rate in our cohort was approximately 1%, substantially lower than rates reported in studies restricted to patients with CAP, in which pneumococcal UAT positivity is substantially higher.^11^ This difference likely reflects, at least in part, the broader testing population in our study, which included all patients undergoing UAT rather than only those meeting clinical criteria for pneumonia. At institutions where *S. pneumoniae* and *Legionella* UATs are routinely ordered together or incorporated into bundled order sets, diagnostic stewardship interventions targeting pneumococcal UAT should consider whether these tests can be addressed independently, given their differing epidemiology and indications for testing.

There are several limitations to our study. First, this was a retrospective, descriptive study and decisions to obtain *S. pneumoniae* UAT were made at the discretion of treating clinicians. Second, the determination of clinically significant infection for positive tests detected later during hospitalization relied on chart review and clinical adjudication, which may introduce subjectivity. Also, it must be emphasized that the test performance will vary across institutions and hosts, and that independent institutional validation is essential prior to implementing stewardship restrictions. Finally, because the cohort was identified based on UAT utilization rather than culture-confirmed pneumococcal infection, we were unable to assess false-negative UAT results.

In summary, the *S. pneumoniae* UAT test yield was low in this oncology cohort including subgroups with neutropenia or preceding cellular therapy. We observed a non-significant decline in test utility after hospital day 3. Our findings support implementation of a diagnostic stewardship approach to the *S. pneumoniae* UAT test among hospitalized cancer patients past hospital day 3. Future studies should prospectively evaluate the impact of diagnostic stewardship interventions restricting late-hospitalization *S. pneumoniae* UAT use on testing rates and clinical outcomes.

## Table 1. Long description

A table with 11 rows and 2 columns summarizing demographics and baseline characteristics of study patients. The columns are labeled ‘Characteristic’ and ‘n (%)’. The table includes data on age groups, sex, inpatient and outpatient status, cancer type, neutropenic status, and treatments like BMT or CAR-T. Notable characteristics include 56 percent of patients aged 65 or older, 53.2 percent male, 90.7 percent inpatients, 31.4 percent with hematologic malignancy, 12.9 percent neutropenic, and 11.4 percent who received BMT or CAR-T.

Navigate back to Table 1..

## Table 2. Long description

A table comparing Streptococcus pneumoniae urinary antigen test positivity rates by patient location and immune status. The table has three rows and four columns. Column headers are Outpatient, Hospital days 0-2, Hospital days >= 3, and P value. Row labels are Positivity rate overall, Positivity rate during the case of neutropenia, and Positivity rate within 1 year of cellular therapy. Row 1: Outpatient, 10/1083 (0.92 percent); Hospital days 0-2, 96/7695 (1.2 percent); Hospital days >= 3, 25/2816 (0.89 percent); P value, 0.15. Row 2: Outpatient, 0/22 (0 percent); Hospital days 0-2, 9/731 (1.2 percent); Hospital days >= 3, 5/738 (0.68 percent); P value, 0.30. Row 3: Outpatient, 1/115 (0.87 percent); Hospital days 0-2, 8/618 (1.3 percent); Hospital days >= 3, 2/586 (0.34 percent); P value, 0.11.

Navigate back to Table 2..
